# Kefiran as a Novel Biomaterial Ink Component: Preliminary Assessment of 3D Printing Feasibility and Biocompatibility

**DOI:** 10.3390/gels12040279

**Published:** 2026-03-26

**Authors:** Elena Utoiu, Andreea Plangu, Vasile-Sorin Manoiu, Elena Iulia Oprita, Rodica Tatia, Claudiu Utoiu, Oana Craciunescu

**Affiliations:** 1 National Institute of Research and Development for Biological Sciences, 060031 Bucharest, Romania; andreea.iosageanu@incdsb.ro (A.P.); sorin.manoiu@incdsb.ro (V.-S.M.); iulia.oprita@incdsb.ro (E.I.O.); rodica.tatia@incdsb.ro (R.T.); oana.craciunescu@incdsb.ro (O.C.); 2 National Research and Development Institute for Food Bioresources—IBA, 020323 Bucharest, Romania; claudiu.utoiu@bioresurse.ro

**Keywords:** kefiran, 3D bioprinting, hydrogel ink, cytotoxicity, antiproliferative activity, tissue engineering

## Abstract

The development of biomimetic scaffolds requires balancing structural integrity with biological signaling. This study evaluates kefiran, a microbial exopolysaccharide, as a bioactive component in establishing printing feasibility of 3D composite constructs. Kefiran from Romanian artisanal cultures was characterized via ^1^H-NMR, HPLC, and SEM/TEM, confirming a high-quality hexasaccharide repeating unit. Three composite inks (K100, K70, and K50) were developed by integrating kefiran, chondroitin sulfate, and Si-substituted hydroxyapatite into an alginate matrix and processed using a Bio X 3D-printer. Results showed that higher kefiran concentrations improved printing feasibility, providing enhanced structural fidelity and stability during the layer-by-layer deposition process. All bioprinted scaffolds demonstrated high cytocompatibility with L929 fibroblasts, maintaining viability above 70%. Notably, kefiran exhibited dual-functional therapeutic potential: concentrations above 500 mg/L showed a concentration-dependent antiproliferative effect against HT-29 cells at 72 h while remaining safe for normal cells. These findings establish kefiran-based biomaterial inks as robust, bioactive platforms for regenerative medicine. By enhancing both the mechanical printability of alginate composites and the biological response of cultured cells, kefiran proves to be a versatile component for advanced tissue engineering and potential biological activity applications.

## 1. Introduction

Kefir grains are composed of microorganisms encapsulated in a polysaccharide matrix. The ratio of the present microbial species varies from one geographic area to another, being composed of *Lactobacillus* sp. (*L. acidophilus*, *L. kefiranum*, *L. kefir*, and *L. parakefir*), *Candida* sp., *Saccharomyces* sp., *Acetobacter* sp., etc. [1]. Kefiran is a high-molecular-weight polysaccharide, secreted by some lactic bacteria present inside the kefir grains, and its main role is to protect microorganisms against dehydration. Kefiran is a water-soluble branched glucogalactan, and it contains D-galactose and D-glucose units in approximately equal quantities [2], being characterized by the repetition of a pentasaccharide unit with one or two monosaccharide branches in random positions in its structure [3,4]. These molecular features are directly linked to kefiran’s functional behavior in aqueous systems (e.g., thickening and gel-forming capacity), which has motivated its exploration as a natural, biocompatible polymer for diverse applications [2,5].

These bacterial exopolysaccharides, because of their status as probiotics for the bacteria that generated them, have become a category of biopolymers that offer new perspectives in the development of products with health and safety benefits [3]. In the last few years, due to its multiple properties, a wide variety of applications were reported in different fields like food packaging (as a biodegradable film) [6,7]; tissue engineering and regenerative medicine [8,9]; biology, due to its prebiotic, antioxidant, and antimicrobial activities [9]; and food industry, where it is explored for use as a biopolymer in edible films and coatings, which offer biodegradability and can enhance shelf life and safety of food products [5,6,7,8,9,10]. Kefiran is also being researched as a postbiotic ingredient for dairy fortification, a carrier for enzyme immobilization, and a possible addition in pharmaceuticals and cosmetics due to its moisturizing and protecting properties. Applications of research are still growing, particularly in the areas of biomedical materials, health-promoting foods, and ecologically friendly packaging.

Beyond techno-functional roles, kefiran and kefir-derived materials have been increasingly investigated for biomedical relevance. Recent work highlights polymer engineering strategies (e.g., oxidation/crosslinking) and composite approaches aimed at producing wound care materials with improved mechanical performance and biocompatibility [8,9], while other reports discuss kefir grain-derived components as bioactive ingredients (including postbiotic-oriented applications) [10]. Although the biomedical landscape for kefiran is expanding, the applications that require simultaneously controlled processability, rapid shape stabilization, and cytocompatibility remain comparatively underexplored.

Kefiran’s diverse therapeutic potential in tissue engineering is driven by several biological mechanisms that work in tandem. Primarily, its antimicrobial activity serves as a critical defense against pathogen colonization, ensuring a stable environment that leads to rapid healing [11]. This is further supported by kefiran’s ability to modulate the local microbiota, suppressing harmful pathogens while maintaining the microbial balance essential for preventing inflammation-driven dysbiosis [9,12].

Beyond infection control, kefiran showed a significant anti-inflammatory effect, minimizing tissue damage and accelerating the transition from the inflammatory phase to the proliferative stage of repair [11]. At a cellular level, it actively stimulates tissue regeneration by promoting collagen synthesis and angiogenesis, which are vital for restoring functional integrity to damaged sites [8,9]. Finally, its film-forming and moisturizing properties enhance barrier function, maintaining optimal hydration and protecting the regenerating tissue from environmental factors [7].

A bibliometric analysis conducted on the Web of Science (WoS) database revealed a growing scientific interest in kefiran, with a total of 258 articles published by early 2025. Many of these studies have emerged within the last 15 years, reflecting an accelerating trend in polysaccharide research. Notably, approximately 28% of the total publications are categorized under Materials Science Composites, Materials Science Biomaterials, Materials Science Multidisciplinary, or Polymer Science. However, despite its promising properties, the specific application of kefiran as a biomaterial or scaffold component in tissue engineering remains an underexplored niche, with only 14 articles addressing this topic in the last 15 years. This scarcity of literature highlights a significant research gap, which implies a lack of clinical trials that test kefiran’s efficacy and safety in human subjects [11], a need to optimize the extraction and production methods of kefiran to enhance its physicochemical properties and bioactivity [13], and a lack of research for potential synergistic effects when combined with other biomaterials that could enhance the mechanical properties and bioactivity of wound dressings [8].

Additionally, more than 4400 references were found in a more general search on the topics of 3D printing and bioprinting, but none of them expressly mentioned kefiran in this technological context. This discrepancy implies that although bioprinting and 3D printing are highly studied and developing topics, the direct use of kefiran in these printing technologies has not yet been well-investigated or recorded.

Extrusion-based 3D bioprinting has emerged as a promising biofabrication route for producing hydrogel-based constructs with controlled architecture for tissue engineering and in vitro models [14,15]. However, the key challenge remains the design of bioinks that can be extruded as continuous filaments while maintaining high shape fidelity (minimizing filament collapse and fusion) without compromising cell viability and function [14,15,16]. alginate-based systems are among the most used platforms due to benign gelation and tunable rheology, yet their performance often requires formulation strategies (blends, nanocomposites, or bioactive fillers) to balance printability, mechanical integrity, and biological performance [17,18]. The development of composite bioinks often relies on the synergy between structural polysaccharides and bioactive glycosaminoglycans to replicate the intricate complexity of natural tissues. While many systems utilize alginate/chitosan, the integration of chondroitin sulfate (CS) plays a critical role in regulating cellular signaling and maintaining tissue hydration, offering a more ‘biologically familiar’ environment for the seeded cells [19,20].

Based on kefiran’s polysaccharide chemistry and emerging evidence of bioactivity, we hypothesized that kefiran can function as a novel ink component for improving the processability and performance of alginate-based composite hydrogel bioinks for extrusion printing. We further considered that adding chondroitin sulfate and silicon-substituted hydroxyapatite may contribute to the structural and biofunctional profile of the printed hydrogels, as suggested by recent nanocomposite bioink developments [18].

In this study, kefiran was extracted from artisanal Romanian milk kefir grains, purified, structurally characterized by HPLC (monosaccharide profiling), FTIR-ATR, and ^1^H NMR-based methods, and biologically evaluated for cytocompatibility and cytotoxic effect in cell cultures. Three alginate-based kefiran composite bioinks were formulated and evaluated for printing feasibility and construct formation, as well as swelling, in vitro stability of the printed scaffolds, and cytocompatibility on L929 fibroblasts. It should be noted that the developed system was specifically optimized for acellular 3D printing and post-printing cell seeding; thus, the cross-linking strategy was tailored to maximize architectural stability and geometric fidelity rather than simultaneous cell encapsulation. By positioning kefiran as an ink component rather than only a bulk hydrogel material, this work aims to expand the toolbox of polysaccharide-based bioinks with potential dual relevance: biofabrication performance and biological functionality.

## 2. Results and Discussion

### 2.1. Preparation and TEM Analysis of Kefiran

**Figure 1:**
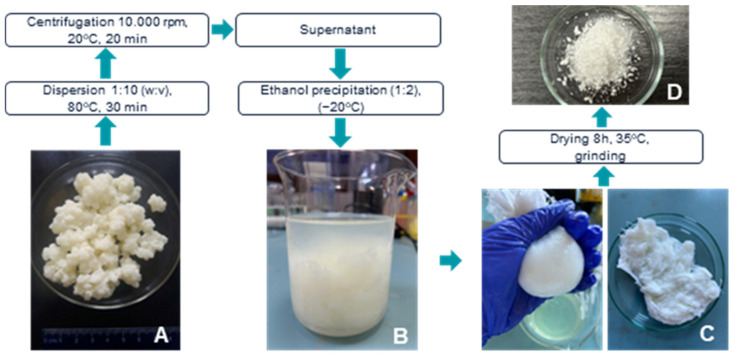
Integrated methodology for the extraction and purification of kefiran from artisanal milk kefir grains. (**A**) Initial artisanal milk kefir grains used as the raw material; (**B**) ethanol precipitation of the supernatant showing the formation of the crude polysaccharide matrix; (**C**) manual recovery and filtration of the precipitated kefiran to isolate the purified biopolymer; (**D**) final conditioned kefiran powder.

Kefiran extraction from Romanian artisanal kefir grains by alcohol precipitation (Figure 1) yielded 17.23 mg/g high-purity biopolymer (approximately 1.7% of kefiran weight). This value aligns with the established literature [21,22]. The resulting yield provided enough refined biopolymer to proceed with the formulation of the K50, K70, and K100 biomaterial inks.

**Figure 2:**
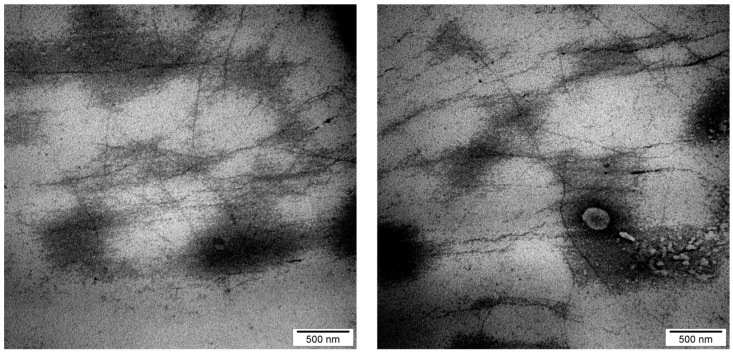
TEM ultrastructural analysis of the branched kefiran network.

The ultrastructural observation (Figure 2) revealed a fibrillar structure of the extracted kefiran with a branched network. The filaments exhibit intermolecular connectivity, which can influence rheological behavior. The distribution of the fibrils is relatively homogeneous, diameters ranging from 6 to 13 nm, with a mean diameter of 9.8 ± 2.5 nm; the measurement were made with the ImageJ (1.54g; Java 1.8.0_355) software, using the scale bar to calibrate the pixel-to-nanometer ratio.

### 2.2. HPLC Analysis of Extracted Kefiran

**Figure 3:**
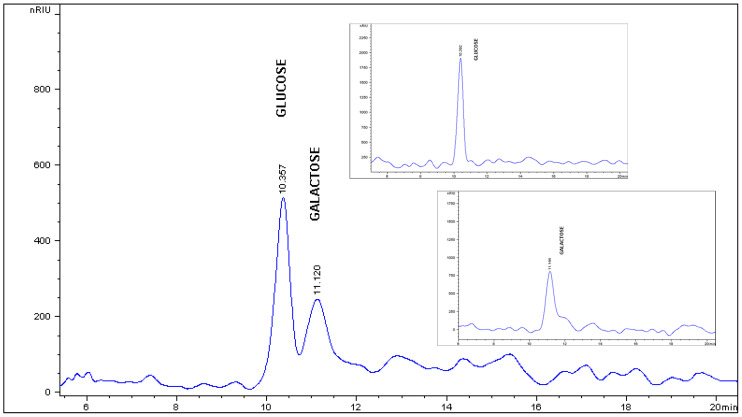
HPLC-RID chromatogram of the hydrolyzed kefiran. The profile illustrates the monosaccharide composition, with distinct peaks identified for glucose (t_R_ = 10.357 min) and galactose (t_R_ = 11.120 min). The *X*-axis represents the retention time (expressed in minutes), and the *Y*-axis represents the refractive index signal (expressed in nano refractive index units, nRIUs). The relative peak areas confirm the glucogalactan nature of the exopolysaccharide.

The HPLC analysis of the monosaccharides profile of kefiran showed D-glucose and D-galactose peaks (Figure 3). The quantitative analysis of the detected monosaccharides showed a ratio of 1:1.08 for glucose:galactose (Table 1). This result agreed with previous studies [4]. Besides glucose and galactose, minor monosaccharides, including arabinose, xylose, and ribose were identified in trace amounts; however, these constituents were not present in sufficient quantities to allow for reliable integration and quantification.

### 2.3. ^1^H-NMR Spectroscopy of Extracted Kefiran

**Figure 4:**
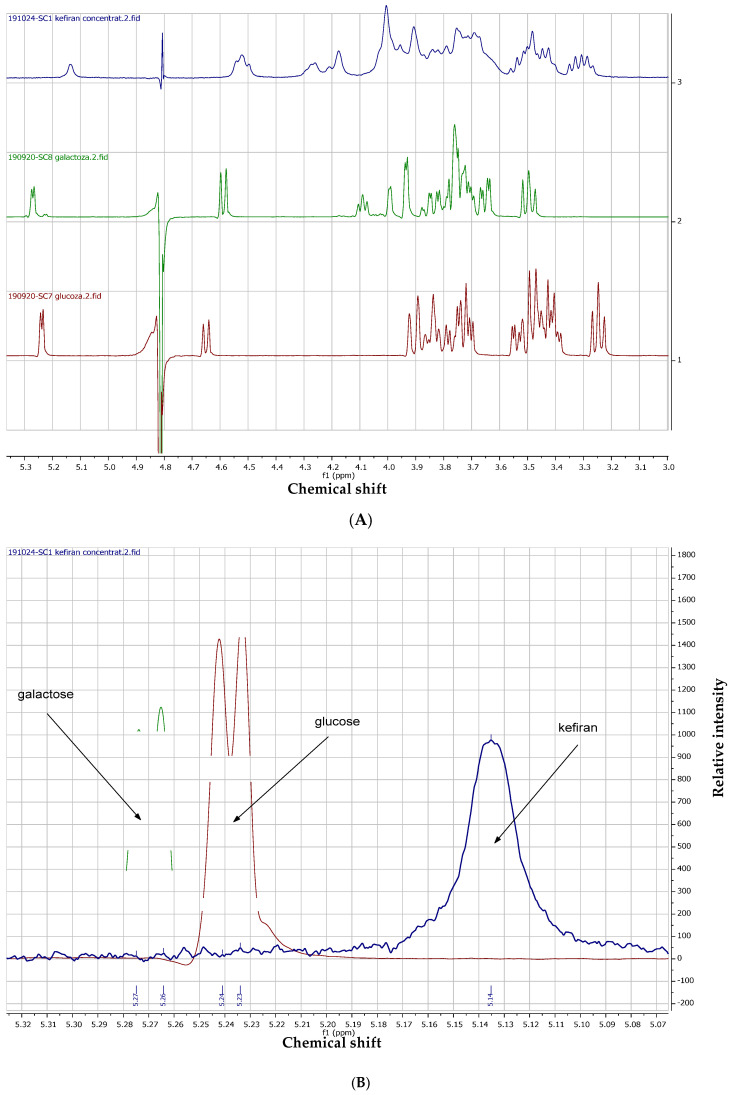
^1^H-NMR spectra of purified kefiran and monosaccharide standards: glucose and galactose; (**A**) comparative stacked plot illustrating the characteristic anomeric proton region; (**B**) detailed view of the chemical shift area between 5.0 and 5.4 ppm.

^1^H-NMR spectroscopy was conducted as a powerful non-destructive tool to elucidate the primary structural features of the extracted kefiran. This technique allows for the precise identification of the anomeric configuration (α or β linkages) and the determination of the glucose-to-galactose molar ratio through peak integration. Furthermore, ^1^H-NMR provides a structural ‘fingerprint’ that confirms the purity and the highly branched nature of the polysaccharide, which are critical factors influencing its physicochemical and biological properties. ^1^H-NMR spectra of kefiran and standards showed the specific singlet at 5.14 ppm, assigned to hydrogen linked to anomeric carbon, in the anomeric area (5.3–4.4 ppm) (Figure 4A). Compared to component monosaccharides of glucose and galactose, which present a doublet in a proximal area, the kefiran sample had a singlet because of polymeric bonds, as can be seen in the detailed graphic representation (Figure 4B).

**Figure 5:**
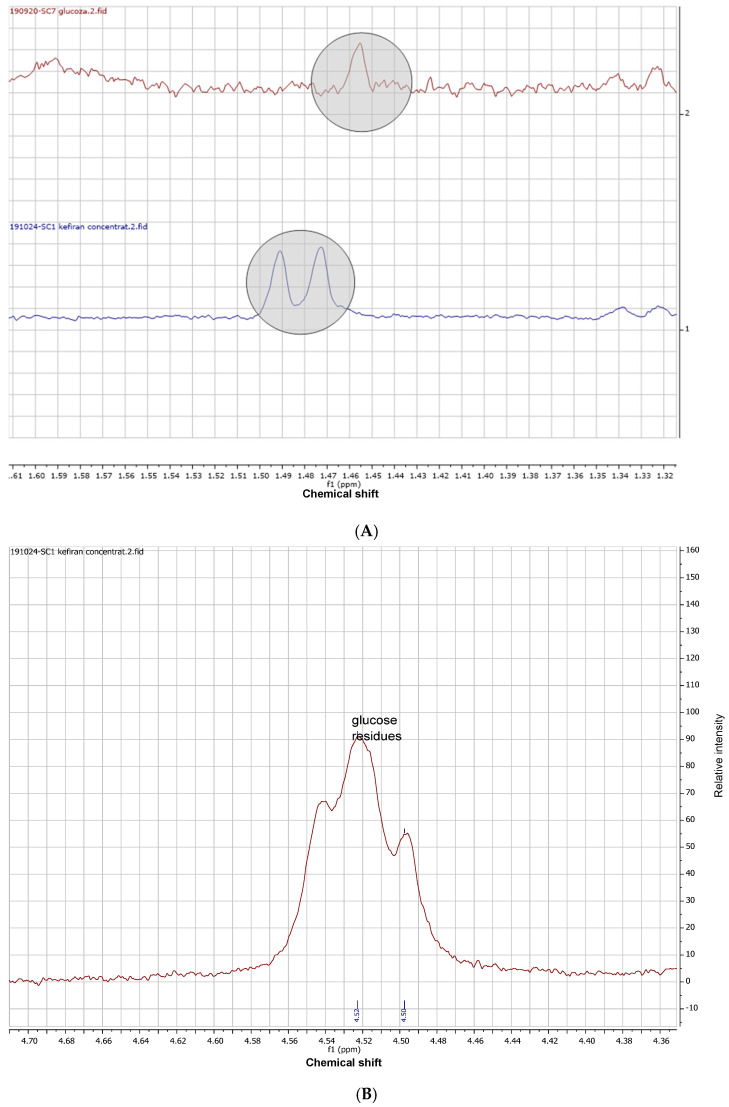
(**A**) ^1^H-NMR spectra of kefiran and glucose in the area of 1.50–1.45 ppm; (**B**) glucose residues at 4.52 ppm.

It was also observed that, in the range of 1.50–1.45 ppm, glucose had a singlet at 1.45 ppm, while kefiran had a doublet at 1.49 and 1.47 ppm, respectively (Figure 5A), confirming that the structural unit of kefiran was closed with a glucose unit. This assignment is specific to a H linked to a C-C-O. Also, glucose residues were observed in the anomeric region of the ^1^H-NMR spectra (Figure 5B).

**Figure 6:**
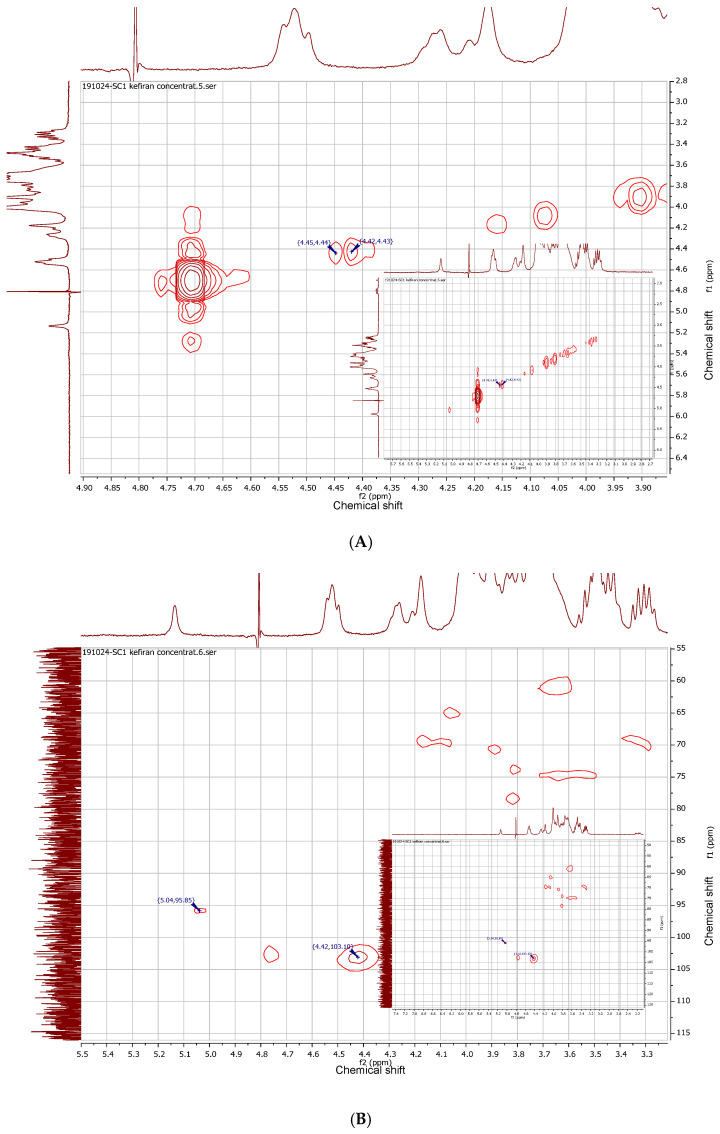
The COSY (**A**) and HMQC (**B**) spectra of kefiran (the top- and side-spectra are the 1D ^1^H-NMR reference projections for the 2D correlation map).

Cross-peaks were observed in the COSY spectra and were confirmed with the HMQC spectra (Figure 6A,B). This was possible to see even at lower concentrations of kefiran in D_2_O.

### 2.4. FTIR-ATR Spectroscopy of Extracted Kefiran

**Figure 7:**
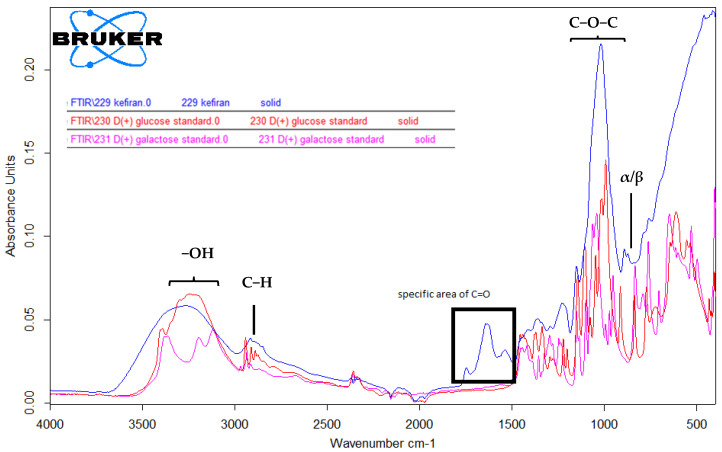
FTIR-ATR spectra of kefiran compared to glucose and galactose standards. The major absorption bands are identified as follows: the broad peak at 3400–3200 cm^−1^ corresponds to the -OH stretching vibrations; the bands at 2900–2800 cm^−1^ relate to C-H stretching; the intense peaks in the 1150–1000 cm^−1^ region are characteristic of the C-O-C glycosidic bridge stretching in the polysaccharide backbone. The highlighted area at 1650–1700 cm^−1^ (C=O) suggests minor contributions from open-chain monosaccharide forms, while the signals at 900–800 cm^−1^ confirm the anomeric configuration of the glucogalactan units.

The structural functional groups of the extracted kefiran and the analytical standards of glucose and galactose were characterized using FTIR-ATR. The FTIR-ATR spectra of kefiran indicated a significant absorption area specific to the C=O (carbonyl) group (Figure 7), suggesting that the sample contained glucose or galactose molecules not integrated into the main structural unit of the biopolymer but existing as free molecules. However, the presence of certain absorption bands, in agreement with the ^1^H-NMR analysis, may suggest the existence of specific structural features within the polysaccharide network. These features could be associated with flexible chain conformations, which may contribute to intermolecular interactions and potential cross-linking effects. As these observations are hypothetical at this stage, a comprehensive analytical confirmation of the full molecular architecture should be addressed in a separate, specialized research study.

The FTIR observations were corroborated by the ^1^H-NMR data, specifically the presence of glucose residues at 4.52 ppm associated with the anomeric carbon. This dual-method approach confirmed the presence of these residues within or alongside the polysaccharide matrix. The rest of the spectra supports the conclusion that kefiran is a biopolymer with structural units made of glucose/galactose.

### 2.5. In Vitro Cytocompatibility Testing and Antiproliferative Activity

**Figure 8:**
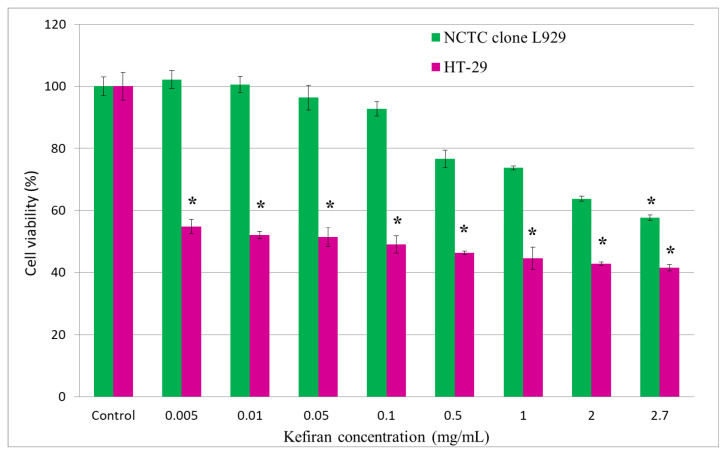
Cell viability of murine fibroblasts, L929, and human colorectal adenocarcinoma cells, HT-29, cultivated in the presence of different kefiran concentrations for 72 h and evaluated by the MTT assay. Data were expressed as mean of three replicates ± SD (*n* = 3). * *p* < 0.05.

The results of the MTT assay in the normal L929 cell line are presented in Figure 8. The data revealed a concentration-dependent effect of kefiran on cell viability. Moreover, kefiran proved to be non-cytotoxic to normal fibroblasts at concentrations up to 1 mg/mL (cell viability > 70%). At low concentrations (0.005–0.01 mg/mL), kefiran exhibited a stimulatory effect on cell proliferation, but the values were not significantly (*p* > 0.05) different from control (100%). A slight cytotoxic effect (58–70% cell viability) was observed at higher concentrations (2–2.7 mg/mL).

Kefiran testing on HT-29 tumor cells showed a marked inhibitory effect on colorectal adenocarcinoma cell viability in a dose-dependent manner. All tested concentrations (0.005–2.7 mg/mL) showed a significant (*p* < 0.05) effect of reducing cell viability down to 40% compared to control (100%) (Figure 8).

**Figure 9:**
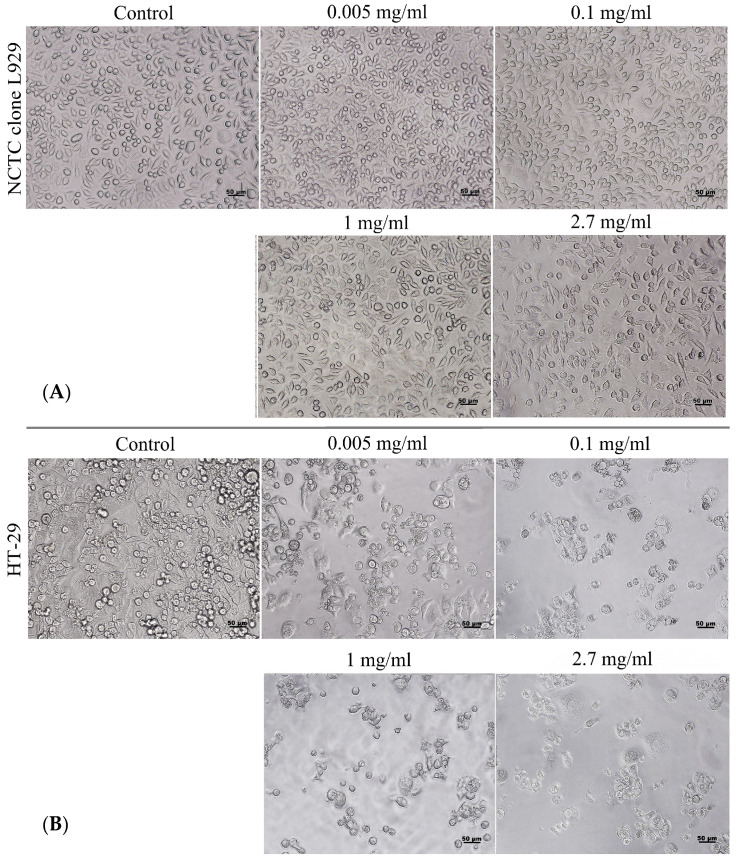
Microscopy images of L929 (**A**) and HT-29 (**B**) cells cultivated in the presence of different kefiran concentrations.

Qualitative observations via inverted microscopy confirmed the quantitative MTT results, showing a cell density similar to that of control and typical morphology of L929 cells in cultures treated with up to 1 mg/mL kefiran (Figure 9). In contrast, tumoral HT-29 cells showed signs of structural distress and decreased density at all tested concentrations, confirming a significant inhibitory effect of kefiran on tumor cell proliferation (Figure 9).

We used the MTT assay as a reliable screening tool for this stage of our research to prove kefiran’s antiproliferative effect, but because these results are strictly preliminary observations, further studies incorporating flow cytometry for cell cycle analysis and oxidative stress markers (ROS) will be necessary to fully understand the molecular mechanisms behind this potential.

### 2.6. Kefiran Composite Hydrogel Bioink Characterization

Kefiran biopolymer was translated into a functional bioink through the optimization of composite formulations in association with CS, Si-substituted hydroxyapatite (Si-HA), and alginate, selected as a stable, biocompatible matrix based on the previously established literature in tissue engineering [18,19,20,21,22,23]. To understand how kefiran influences the printing process, we evaluated the structural fidelity of these kefiran-augmented inks by considering the intrinsic viscosity of the biopolymer. The results of intrinsic viscosity of non-cross-linked kefiran bioinks demonstrated a clear linear correlation to kefiran concentration, presenting a value of 9.8 dL/g for K100 and 6.7 dL/g for K50 (Table 2).

The higher intrinsic viscosity of K100 (9.79 dL/g) compared to K50 (6.67 dL/g) suggests a more robust macromolecular network, which could potentially contribute to the structural stability of the ink during the 3D printing process. While intrinsic viscosity is a molecular parameter rather than a direct printability metric, these values provide a preliminary indication of the polymer’s ability to influence the consistency of the composite formulation.

A direct link between these results and the values of swelling degree was observed. K100 bioink, having the highest intrinsic viscosity, demonstrated the highest water-binding capacity (85.16%). These results indicated that the larger molecular network of K100 could effectively trap and hold more water molecules within its structure.

### 2.7. 3D Bioprinted Structure Characterization

**Figure 10:**
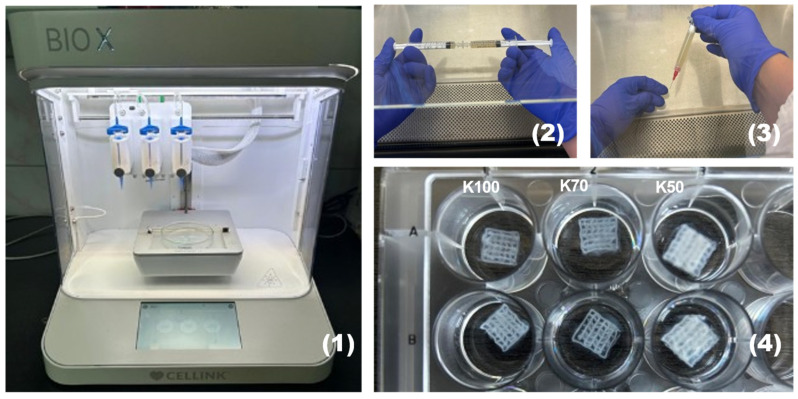
Fabrication of kefiran 3D-printed constructs. (**1**) The 3D bioprinter Bio X (Cellink, Brighton, UK) used for the layer-by-layer extrusion of hydrogel bio-ink; (**2**) Homogenization and loading of the bio-ink formulations into 3 mL cartridges; (**3**) Precision extrusion using a 25G (0.25 mm) metal tip; (**4**) Representative images of the 3D bioprinted grid-like constructs (10 × 10 × 0.5 mm, 36 internal grids), denoted K100, K70, and K50 depending on kefiran concentration.

All three bioink formulations (K100, K70, and K50) were used to print 3D grid-like constructs (Figure 10).

The gravimetric analysis showed that all cross-linked kefiran 3D-printed hydrogel bioink formulations (K100, K70, and K50) had strong water-binding capacities. Most of the swelling happened within the first 2 h of immersion (Table 3).

The performance of the scaffolds is further defined by their swelling and degradation profiles, which govern the stability of the construct in a physiological-like environment. The controlled degradation behavior, paired with the internal diffusion capacity of the hydrogel, ensures a steady environment for cell growth. The incorporation of Si-HA was intended to enhance the general regenerative potential of the matrix, aligning with current trends in mineralized scaffolds that aim to provide both structural support and essential inorganic cues for unspecified tissue regeneration [24,25].

The obtained data suggested that the scaffolds reached near-equilibrium state quickly. A clear pattern emerged: the swelling degree increased as the kefiran content went up. K100, which had the highest kefiran concentration, displayed the greatest swelling capacity, reaching 85.16 ± 3.01% after 24 h. This rapid hydration is beneficial for 3D bioprinting applications, as it ensures that the scaffold quickly stabilizes its volume and internal osmotic pressure upon contact with the culture media. K50 showed the lowest swelling values of 72.65 ± 2.70%. This indicated that increasing kefiran, a highly branched water-soluble glucogalactan (as confirmed by ^1^H-NMR), improved the hydrophilic nature of kefiran/CS/Si-HA in alginate matrix. This allows for better expansion of the polymeric network.

The results of testing in vitro stability of the 3D-printed constructs indicated a controlled, concentration-dependent degradation profile (Table 4).

All formulations maintained their structural integrity over the first 24 h, with mass loss values ranging from ~14.8% to 16.2%. At 72 h, a more pronounced degradation rate (32.52%) for K100 compared to K50 (15.69%) was observed. This implies that K100 creates a complex, highly branched network structure (which explains the increased initial swelling), but it might also have a more dynamic design that could explain the gradual dissolution or hydrolytic disintegration of the kefiran-rich domains.

**Figure 11:**
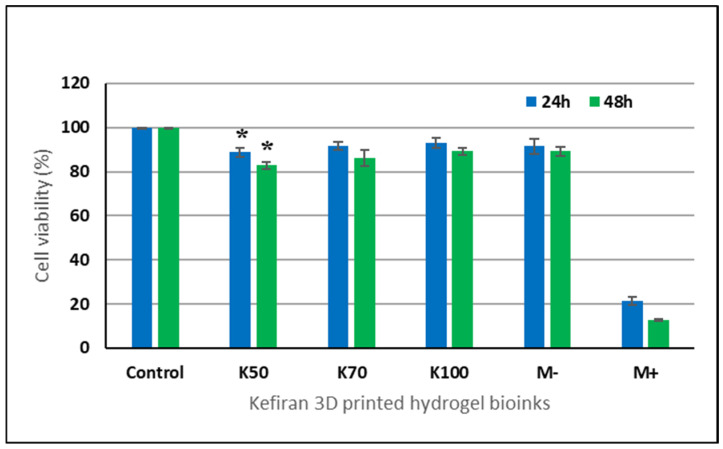
Cell viability of L929 fibroblasts cultivated in the presence of kefiran 3D-printed hydrogel bioinks at 24 and 48 h, determined by MTT assay. Data are presented as mean ± standard deviation (*n* = 3). * *p* < 0.05.

In vitro testing showed that all three tested kefiran 3D-printed hydrogel bioinks (K50, K70, and K100) exhibited a non-cytotoxic effect on L929 fibroblasts, at both 24 and 48 h of cultivation, presenting cell viability values ranging between 82.85% and 92.95% (Figure 11). Among the samples, K100 demonstrated the highest cell viability values of 92.95% at 24 h and 89.26% after 48 h in L929 cells. These results confirmed the biological safety of the newly developed kefiran bioinks for 3D bioprinting applications.

The bioprinted constructs developed in this study are fully hydrated hydrogels rather than lyophilized (freeze-dried) porous scaffolds, and they are characterized by a continuous, water-saturated polymeric network. For this type of materials, instead of a traditional microporous system, the transport of nutrients and metabolic waste occurs via diffusion through the hydrated interstitial spaces of the polymer matrix with a direct impact on cell viability and degradation.

**Figure 12:**
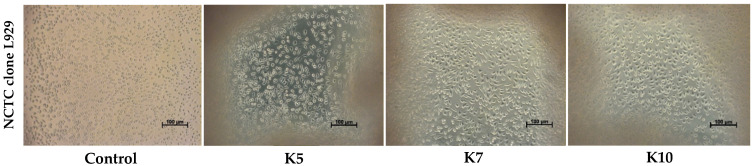
Cell morphology of L929 cells cultivated in direct contact with kefiran 3D-printed hydrogel bioinks (K50, K70, and K100) at 48 h (scale bar = 100 µm). The refractive boundaries visible in the composite samples indicate the cross-linked hydrogel margins.

The cell morphology observations showed that all three stabilized kefiran bioinks (K50, K70, and K100) supported healthy cellular attachment without signs of structural distress or cytotoxicity. Notably, the K100 scaffolds exhibited a higher density of adhered cell, confirming its superior biocompatibility, in accordance with quantitative MTT results (Figure 12).

The biocompatibility results were well-correlated to a previously discussed swelling degree of kefiran bioinks formulation. In case of K100 bioink, superior values of both cytocompatibility in L929 fibroblasts (92.95% at 24 h) and water-binding capacity (85.16% at 24 h) were observed. A higher swelling degree often indicates a more porous and hydrated network, which likely facilitated better cell adhesion and higher viability.

Improved bioactivity of K100 bioink was correlated with a faster rate of disintegration after 48 h; a more dynamic hydrogel matrix frequently promotes better nutrition exchange and cell–matrix interactions, which supports the high viability.

**Figure 13:**
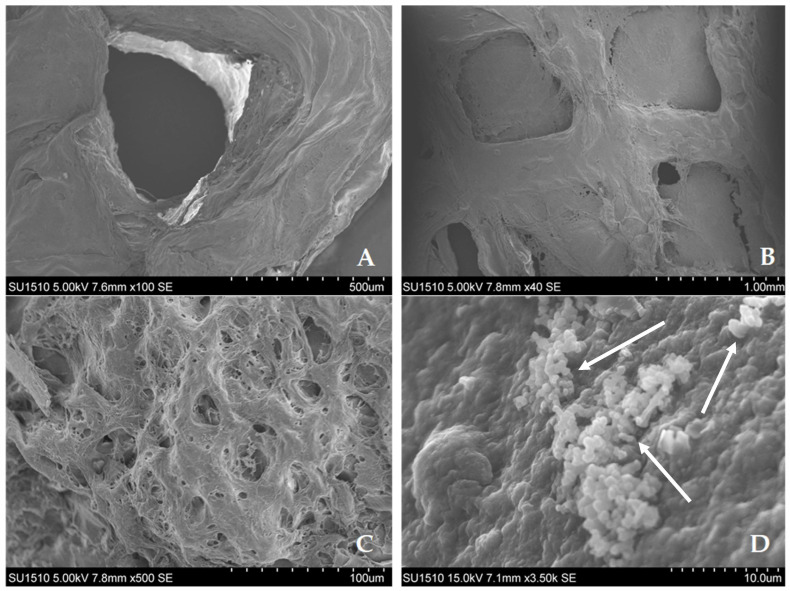
Structural assessment of kefiran-based constructs. (**A**–**C**) SEM surface topography illustrating the transition from high structural integrity in K100 to increased micro-porosity and macro-scale collapse in K50; (**D**) SEM visualization of L929 fibroblast adhesion on the surface of the bioprinted grid-like scaffold (arrows point to cellular anchoring points).

Out of all the examined groups, the K100 sample’s SEM micrographs show the best structural fidelity (Figure 13A). The grid eyes are open and plainly visible, and the grid design is still well-defined. The K100 3D-printed structure, which has a greater kefiran content and higher intrinsic viscosity, has successfully resisted collapse and gravity flow throughout the printing and subsequent cross-linking processes, thereby showing a better stability. For the K70 prints, a change in the structural integrity was observed (Figure 13B).

The filaments show some spreading while maintaining the grid pattern, resulting in partially “collapsed” margins that start to block the grid apertures. The SEM investigation of the K50 scaffold showed a significant collapse of the macro-architecture and a porous microstructure (Figure 13C). This topography is caused by a graduated alcoholic dehydration procedure necessary for the stability of the SEM samples. Even though this led to a compromised macro-scale fidelity, this microporosity indicates a highly permeable environment that promotes quick nutrition exchange and molecular diffusion.

To emphasize the ability of kefiran 3D-printed constructs to support cell attachment on their surface, their cultivation was carried out on the surface of the printed support for 24 h, and the SEM investigation demonstrated this capacity (Figure 13D). The SEM observations showed that NCTC cells were present as cell aggregates on the surface of 3D-printed composite scaffolds after 24 h of incubation.

For the SEM morphological analysis, the samples were chemically fixed and dehydrated through graded ethanol baths prior to imaging, as required for high-vacuum SEM conditions. Therefore, the micrographs represent a fixed snapshot of cell morphology and attachment to the kefiran-based scaffold rather than living cells during imaging. The presence of cellular clusters integrated within the scaffold’s rugose surface provides qualitative evidence of cell adhesion and interaction with the kefiran matrix.

At this preliminary stage, structural fidelity was evaluated qualitatively by examining the consistency of the printed grid pores across the different formulations. The K100 formulation showed the most stable deposition, producing well-defined and regular geometries, whereas the K50 formulation exhibited a greater degree of filament spreading. Although a quantitative morphometric analysis was beyond the scope of this initial biological validation study, these observations nevertheless indicate a clear concentration-dependent effect of kefiran on the printing feasibility of the composite bioinks.

Although the present characterization mainly focuses on intrinsic viscosity and the macroscopic printing fidelity of the formulations, we acknowledge that a more comprehensive rheological evaluation would be beneficial. Future investigations should therefore include oscillatory rheological measurements, such as shear-thinning behavior, yield stress determination, thixotropic recovery, and the analysis of storage and loss moduli (G′, G″), to more fully describe the viscoelastic window of kefiran-based composite systems. Nevertheless, the results obtained in this preliminary study provide encouraging evidence supporting the potential use of kefiran as a component of bioinks for 3D printing constructs.

## 3. Conclusions

This study successfully demonstrated the extraction and structural characterization of a high-purity kefiran biopolymer from artisanal milk kefir grains.

The ^1^H-NMR and FTIR-ATR spectroscopic analysis confirmed the isolation of a highly branched water-soluble glucogalactan. The identification of a specific anomeric singlet at 5.14 ppm and the terminal glucose doublet at 1.49/1.47 ppm provided a clear structural fingerprint of the polysaccharide. Furthermore, the correlation between FTIR carbonyl signals and the ^1^H-NMR peak at 4.52 ppm offered evidence of a complex matrix containing integrated glucose residues and potential non-cyclic sugar forms.

The translation of this biopolymer into a functional bioink was achieved by the optimization of composite formulations in association with CS, Si-HA, and alginate. Among the tested formulations, K100 emerged as the superior candidate for tissue engineering applications. Its performance was characterized by high hydration capacity, reaching a maximum swelling degree of 85.16%, which facilitated efficient nutrient diffusion and demonstrated excellent biocompatibility, supporting the highest viability of NCTC fibroblasts (92.95% at 24 h).

In conclusion, the K100 formulation represents a promising, safe bioink, bridging the gap between artisanal natural resources and advanced 3D bioprinting technologies for regenerative medicine.

By combining the structural purity confirmed by ^1^H-NMR with optimized printing parameters and rheological performance, K100 was identified as the most effective formulation. It offers a good balance between the viscosity requirements of 3D bioprinting and the biological requirements for supporting healthy cell morphology and proliferation.

This study provides initial insights into the potential use of kefiran as a bioactive component in composite hydrogel bioinks for extrusion-based 3D bioprinting. The incorporation of kefiran into alginate-based formulations resulted in an encouraging printing feasibility and supported cytocompatibility under the evaluated conditions. The present work primarily examined the swelling behavior and structural stability, which offer an initial indication of how the hydrogel may perform under physiologically relevant conditions. However, further studies—including compressive and tensile mechanical testing—will be necessary to better define the mechanical properties of these scaffolds and relate them to the requirements of specific tissues. Given the preliminary nature of this study, additional research is also required to optimize the formulation parameters and to further investigate the long-term biological and rheological performance of these systems. Nevertheless, these findings provide a proof of concept supporting the potential of kefiran-containing composite hydrogels as bioactive scaffolds for tissue engineering and regenerative medicine.

## 4. Materials and Methods

### 4.1. Kefiran Extraction

Kefiran extraction was performed from lyophilized artisanal milk kefir grains, following the protocol described by Rimada and Abraham (2003) [26], with slight modifications [27]. The grains were finely triturated and dispersed in distilled water at 80 °C under continuous stirring for 30 min, using a 1:10 (*w*/*v*) ratio. After centrifugation at 10,000 rpm and 20 °C for 20 min, the kefiran was isolated from the supernatant through three successive ethanol precipitations (*v*/*v*, 1:2) using cold chilled ethanol (−20 °C). The initial precipitate was maintained overnight at −20 °C before proceeding with the subsequent precipitation steps. Each stage was followed by centrifugation (10,000 rpm, 4 °C, 20 min) and redissolution of the pellet in water at 60 °C. The resulting samples (Figure 1) were either stored as pellets in a freezer or conditioned as a powder by drying for 8 h at 35 °C, followed by grinding of the resulting material.

### 4.2. Ultrastructural Analysis by Transmission Electron Microscopy (TEM)

The morphological and ultrastructural characteristics of the extracted kefiran were evaluated using a EM 208S transmission electron microscope (Philips-FEI, Hillsboro, OR, USA), equipped with a Veleta video camera (Olympus Soft Imaging Solutions GmbH, Munster, Germany) and the iTEM Olympus Soft Imaging software version 1224 [27]. The kefiran sample was fixed in a glutaraldehyde–paraformaldehyde solution (3%:1.5% in 1 M Na_3_PO_4_) for 2 h, followed by post-fixation in 1% OsO_4_ and 0.5 M Na_3_PO_4_ for 1 h in the dark. Serial ethanol concentrations (12.5% to 100%) were used for dehydration, followed by gradual infiltration with acetone–resin mixtures (1:1 and 1:2 ratios) and final embedding in pure resin. Polymerization was performed at 50 °C for 60 h. Ultrafine sections were obtained using a UC6 ultramicrotome (Leica, Wetzlar, Germany) with a diamond knife and collected on 200-mesh Formvar-coated copper grids. Double-contrast staining was achieved using 5% uranyl acetate in methanol (7 min) and a lead citrate/trisodium citrate solution (7 min) before imaging.

### 4.3. HPLC Analysis

Acid hydrolyzed samples of kefiran (10 mg) were prepared by incubation in 4 M trifluoroacetic acid (1 mL) at 115 °C for 120 min [27,28]. The resulting reaction mixture was centrifuged at 10,000× *g* for 10 min and dried at low pressure at 90 °C. The samples were washed twice with methanol to remove traces of trifluoroacetic acid and evaporated at low pressure at 90 °C. After the evaporation, the samples were redissolved in 1 mL pure water.

The identification and quantification of monosaccharides derived from kefiran was conducted using an Agilent 1200 HPLC system (Agilent Technologies, Santa Clara, CA, USA) equipped with an online degasser, isocratic pump, autosampler, and a refractive index detector (RID). Chromatographic separation was achieved using a ZORBAX carbohydrate analysis column (Φ 4.6 × 150 mm). The analysis was performed with 75% acetonitrile at 0.5 mL/min flow rate, 35 °C optical unit temperature, 30 °C column temperature, and a 5 μL injection volume. Calibration curves were built using a monosaccharide kit (47267, Supelco, Bellefonte, PA, USA), from which standard stock solution containing D(−)arabinose, D(−) fructose, D(+)galactose, D(+)glucose, D(−)ribose, D(+)mannose, and D(+)xylose were prepared, with a concentration range from 0.5 to 1.5 mg/mL. Data acquisition and processing were performed using the Agilent ChemStation software (version B 03.01 SR1).

### 4.4. ^1^H-NMR Spectroscopy

The powder of extracted kefiran was dissolved in D_2_O (min. 99.95%) with internal reference of TSP 52 mg/100 mL in a ratio of 2:1 (*v*/*v*) for chemical shift calibration (δ = 0.00 ppm). In parallel, the analytical standards of D-glucose and D-galactose were analyzed under the same experimental conditions to serve as references for the assignment of the NMR signals [22]. The samples were analyzed in 5 mm NMR tubes (Wilmad, Vineland, NJ, USA). The ^1^H-NMR spectra were run on a Avance III, 400 MHz spectrometer (Bruker, Billerica, MA, USA), operating in a 9.4T electromagnetic field, corresponding to a resonance frequency of 400.13 MHz for ^1^H nucleus. ^1^H-NMR spectra were recorded at a 450-pulse sequence without attenuated power, 2.05 s acquisition time, 6.4 kHz spectral window, 16–128 scans, 26 k data points, and 1 s relaxation time. The ^1^H-NMR spectra acquisition with IconNMR version (Bruker, Billerica, MA, USA) was conducted for 2 min. All spectra were processed with TopSpin 3.6.x version and Mnova 14.

### 4.5. Fourier Transform Infrared Spectroscopy in Attenuated Total Reflectance Mode (FTIR-ATR)

The FTIR spectra were recorded using a Invenio S spectrometer (Bruker, Billerica, MA, USA) equipped with a horizontal Attenuated Total Reflectance (ATR) accessory and a diamond crystal [22]. The analysis was performed directly on solid kefiran without prior preparation. The spectra were acquired in the spectral range of 4000–550 cm^−1^ with a resolution of 4 cm^−1^. For each measurement, 64 scans were performed for both the sample and the background. Data acquisition and processing were assisted by the OPUS 8.2.8 software.

### 4.6. In Vitro Cytocompatibility Testing

The cytocompatibility of the extracted kefiran and three distinct kefiran biomaterial ink formulations (prepared as described below) was evaluated in a normal stabilized cell line NCTC clone L929 (murine fibroblasts, normal cells), using direct contact method according to the international standard for medical devices, ISO 10993/5 [29], to determine their suitability for tissue engineering applications. For the experiment, L929 cells were cultivated in Minimum Essential Medium (MEM), supplemented with 10% Fetal Bovine Serum (FBS) and 1% penicillin/streptomycin/neomycin (PSN) mix. Subconfluent cells were trypsinized and seeded into 96-well plates at a density of 4 × 105 cells/mL, and plates were incubated at 37 °C in a humidified atmosphere with 5% CO_2_ for 24 h to allow cell attachment. Following the adhesion, the medium was replaced with fresh culture medium containing different concentrations of kefiran or kefiran biomaterial ink formulations, and the cells were cultured for 72 h under standard conditions. At the end of the incubation period, cell viability was assessed using the 3-(4,5-dimethylthiazol-2-yl)-2,5-diphenyltetrazolium bromide (MTT) assay, as previously described [27,30]. The culture medium was replaced with an equal volume of the MTT solution (0.25 mg/mL) and incubated at 37 °C for 3 h. The resulting formazan crystals were dissolved in isopropyl alcohol under agitation at room temperature for 15 min, and the absorbance was recorded at 570 nm using SPECTROstar^®^ Nano microplate reader (BMG LabTech GmbH, Ortenberg, Germany). The results were normalized against the control group (untreated culture), considered 100% viable, and reported as mean ± SD from triplicate experiments.

Cell morphology was qualitatively assessed in a parallel experiment under the conditions identical to those described above. At the end of the incubation period, the cells were photographed using an inversed microscope with a 20× objective (Carl-Zeiss, Oberkochen, Germany).

### 4.7. In Vitro Cytotoxic Effect

Kefiran cytotoxic effect was evaluated in tumoral HT-29 cell line (human colorectal adenocarcinoma) cultured in Dulbecco’s Modified Eagle Medium (DMEM), supplemented with 10% Fetal Bovine Serum (FBS), 1% penicillin/streptomycin/neomycin (PSN) mix, and 1% Glutamax [27]. For the experiment, HT-29 cells were seeded into 96-well plates at a density of 106 cells/mL, and plates were incubated at 37 °C in a humidified atmosphere with 5% CO_2_ for 24 h. The cell treatment was conducted as described above, followed by the MTT assessment for cell viability.

### 4.8. Preparation of Kefiran Composite Hydrogel Bioinks

Three distinct composite hydrogel bioink formulations (K-ink) were developed using a base of sodium alginate supplemented with chondroitin sulfate (CS) (from bovine trachea, C9819 Sigma-Aldrich, Saint Louis, MO, USA), Si-substituted hydroxyapatite (Si-HA) (kindly donated by SC CHEMI CERAMIC F SRL, Sf Gheorghe, Romania), and different concentrations of kefiran (50, 70, 100 mg/mL). The compositions, denoted as K100, K70, and K50, were prepared in the following mass ratios—kefiran:CS:Si-HA = 10:1:1 (*w*/*w*), kefiran:CS:Si-HA = 7:1:1 (*w*/*w*), and kefiran:CS:Si-HA = 5:1:1 (*w*/*w*)—in alginate (180947 Sigma-Aldrich, with viscosity 15–25 cps) base with a final concentration of 8% (*w*/*v*). All constituents were homogenized in the alginate base on a magnetic stirrer at 40 °C, 100 rpm for 2 h, followed by centrifugation for gas bubble release for 10 min, 5000× *g*, at room temperature.

As this is a preliminary study, our approach was to maintain the concentrations of the inorganic phase (Si-HA) and the primary structural polymers (CS and alginate) constant, while systematically varying the concentration of kefiran (K100, K70, and K50). These variations served as the primary optimization path for translating the biopolymer into a functional, printable bioink.

### 4.9. Determination of Bioink Viscosity

The intrinsic viscosity [η] of a polymer is a direct reflection of its molecular weight and hydrodynamic volume in a specific solvent. The intrinsic viscosity ([η], dL/g) of the kefiran bio-inks was determined by the measurements of the diluted solutions in distilled water at 37 °C, using an Ubbelohde viscometer in a VB 1423 thermostated water bath (Selecta, Madrid, Spain). Four measurements were taken for each type of sample. The results were expressed as mean of the four determinations ± standard deviation (SD).

### 4.10. Kefiran 3D Hydrogel Bioprinting and Cross-Linking

The 3D printing was conducted using an acellular approach. 3D-printed constructs with controlled porosity were fabricated from kefiran bioinks using a Bio X 3D printer (Cellink, Brighton, UK) via a layer-by-layer extrusion technique. Grid-like structures (10 × 10 × 0.5 mm, 36 internal grids) consisting of two layers were printed using 3 mL cartridges equipped with 25G (0.25 mm) metal tips (Figure 10). To ensure high structural fidelity, the printing process was conducted at a speed of 2.2 mm/s with an extrusion pressure of 180 kPa, while the print bed temperature was maintained at 20 °C. The structural integrity of the printed constructs was ensured through a two-step cross-linking procedure that locked the multi-component architecture in place. Primary cross-linking took place at the beginning by structures printing directly into a 50% ethanol bath. This triggered an immediate solidification of the kefiran in alginate, effectively “freezing” the filament’s shape right after extrusion. Secondary cross-linking involved immersing the printed constructs in a solution of 2% CaCl_2_ and 50% ethanol at room temperature for 2 h to complete the ionic gelation of the alginate matrix. Following the cross-linking, 3D-printed constructs were washed 10× with ultrapure water to ensure the complete removal of residual alcohol. The sterilization was performed in a dedicated chamber using UV light exposure for 4 h on each side prior to cell seeding and biocompatibility testing. Biocompatibility was subsequently evaluated by seeding cells onto the surface of the prepared scaffolds, thus avoiding direct cellular exposure to the cross-linking solvents.

### 4.11. 3D Constructs Characterization

#### 4.11.1. Determination of Swelling Degree for Kefiran 3D Printed Constructs

The swelling degree is a critical parameter for 3D-printed hydrogel constructs, as it influences the printability and mechanical stability of the 3D-printed construct in a physiological environment. The determination of the swelling degree was carried out through a gravimetric method [31]. The dried samples were initially weighed (*W_i_*) and placed in ultrapure water at room temperature for 24 h. The hydrated structures were removed from the liquid, gently dried on filter paper, and reweighed (*W_f_*). The swelling degree was calculated as the percentage of water absorption, relative to the initial weight of the dried sample, according to the equation:

$$Degree\;of\;swelling\;(\%)=\frac{\left(W_{f}-W_{i}\right)}{W_{i}}\times 100$$

#### 4.11.2. In Vitro Stability and Mass Loss Analysis for 3D Printed Constructs

The structural stability of the printed and reticulated composite hydrogels under simulated physiological conditions was evaluated via a gravimetric mass loss assay [32]. The dried scaffold samples of known initial weight (*W_i_*) were immersed in phosphate-buffered saline (PBS, pH 7.4) and incubated at 37 °C for 24, 48, and 72 h. At each time point, the samples were removed, dried to constant weight, and reweighed to obtain the final dry mass (*W_f_*). The degradation degree, representing the weight loss percentage, was calculated using the following equation:

$$Weight\;loss\;(\%)=\frac{\left(W_{i}-W_{f}\right)}{W_{i}}\times 100$$

#### 4.11.3. Scanning Electron Microscopy (SEM)

For structural assessment of kefiran 3D-printed hydrogel bioinks (K100, K70, and K50) and a K100 printed structure with cells attached on the surface, a Hitachi SU-1510 scanning electron microscope (SEM) was used. For sample stabilization, serial ethanol concentrations (12.5% to 100%) were used for dehydration. After dehydration, samples were mounted on stubs and sputter-coated with a thin gold layer to ensure electrical conductivity and high-resolution imaging of the scaffold’s fibrillar matrix.

### 4.12. Statistical Analysis

All experiments were performed in triplicate (*n* = 3). The results are expressed as mean ± standard deviation (SD). The statistical analysis was carried out using one-way analysis of variance (ANOVA) and Student’s *t*-test for pairwise comparisons between the control and treated samples. The differences were considered statistically significant at *p* < 0.05.

## Figures and Tables

**Table 1:** Quantification of monosaccharides in kefiran.

**Kefiran**	**Quantification of Compounds by HPLC (µg/mg Kefiran)**						
	D(−) Ribose	D-(+)-Xylose	D(−) Arabinose	D(−) Fructose	D(+) Manose	D-(+)-Glucose	D(+) Galactose
	Tr	Tr	Tr	ND	ND	15.7865	17.0293

**Tr**: trace amounts detected, but not quantified; ND: not detected.

**Table 2:** Intrinsic viscosity values of kefiran bioink determined at 37 °C.

Sample	Intrinsic Viscosity [η] (dL/g) *	Significance
K100	9.79 ± 0.57	Superior capacity for maintaining structural fidelity post-extrusion.
K70	7.75 ± 0.44	Intermediate flow resistance.
K50	6.67 ± 0.38	Lowest viscosity; potentially higher diffusion rates.

* Data are presented as mean ± standard deviation (*n* = 3).

**Table 3:** Swelling degree of different kefiran 3D-printed constructs at 2 and 24 h of incubation.

Sample	Degree of Swelling at 2 h * (%)	Degree of Swelling at 24 h * (%)
K100	80.99 ± 2.55	85.16 ± 3.01
K70	79.69 ± 2.61	83.20 ± 2.45
K50	71.40 ± 2.23	72.65 ± 2.70

* Data are presented as mean ± standard deviation (*n* = 3).

**Table 4:** Degradation profile of kefiran 3D-printed constructs.

Sample	Mass Loss at 24 h * (%)	Mass Loss at 48 h * (%)	Mass Loss at 72 h * (%)
K100	16.17 ± 0.24	28.78 ± 0.49	32.52 ± 0.55
K70	15.84 ± 0.17	22.70 ± 0.36	24.04 ± 0.38
K50	13.89 ± 0.18	14.79 ± 0.20	15.69 ± 0.19

* Data are presented as mean ± standard deviation (*n* = 3).

## Data Availability

The original contributions presented in this study are included in the article. Further inquiries can be directed to the corresponding author.

## References

[1] Nejati F., Junne S., Neubauer P. (2020). A Big World in Small Grain: A Review of Natural Milk Kefir Starters. Microorganisms.

[2] Wang Y., Ahmed Z., Feng W., Li C., Song S. (2008). Physicochemical properties of exopolysaccharide produced by *Lactobacillus kefiranofaciens* ZW3 isolated from Tibet kefir. Int. J. Biol. Macromol..

[3] Kooiman P. (1968). The chemical structure of kefiran, the water-soluble polysaccharide of the kefir grain. Carbohydr. Res..

[4] Micheli L., Uccelletti D., Palleschi C., Crescenzi V. (1999). Isolation and characterisation of a ropy *Lactobacillus* strain producing the exopolysaccharide kefiran. Appl. Microbiol. Biotechnol..

[5] Gentry B., Cazón P., O’Brien K. (2023). A comprehensive review of the production, beneficial properties, and applications of kefiran, the kefir grain exopolysaccharide. Int. Dairy J..

[6] Bodea I.M., Muste A., Cătunescu G.M., Mureşan C. (2017). Bacterial biofilms as wound healing dressing—A review. Sci. Works C Vet. Med..

[7] de Oliveira J.G., Duarte L.G.R., Belo L., de Souza T.F.C., Bitencourt A.H., Yamim S.T., Egea M.B. (2025). Recent advances in kefiran polymer to produce nanofibers and films for food packaging applications. Food Humanit..

[8] Chelminiak-Dudkiewicz D., Machacek M., Dlugaszewska J., Mylkie K., Smolarkiewicz-Wyczachowski A., Kozlikova M., Druzynski S., Krygier R., Ziegler-Borowska M. (2025). The role of dialdehyde kefiran as a crosslinking agent in chitosan/kefiran-based materials with “Henola” extracts: A biocompatible strategy in wound care. Mater. Adv..

[9] Xu H.T., Li Y.Q., Song J.P., Zhou L.Y., Wu K.Z., Lu X.Y., Zhai X.N., Wan Z.L., Gao J. (2024). Highly active probiotic hydrogels matrixed on bacterial EPS accelerate wound healing via maintaining stable skin microbiota and reducing inflammation. Bioact. Mater..

[10] Coban F., Özer E.D., Yildirim M. (2025). A new approach to the use of kefir grain: Potential application as a postbiotic in the production of nonfat yogurt. Food Sci. Nutr..

[11] Huseini H.F., Rahimzadeh G., Reza Fazeli M., Mehrazma M., Salehi M. (2012). Evaluation of wound healing activities of kefir products. Burns.

[12] Arshad T., Mundrathi V., Perez V.E., Nunez J.M., Cho H. (2024). Topical Probiotic Hydrogels for Burn Wound Healing. Gels.

[13] Hasheminya S.M., Dehghannya J. (2020). Novel ultrasound-assisted extraction of kefiran biomaterial, a prebiotic exopolysaccharide, and investigation of its physicochemical, antioxidant and antimicrobial properties. Mater. Chem. Phys..

[14] Murphy S.V., Atala A. (2014). 3D bioprinting of tissues and organs. Nat. Biotechnol..

[15] Schwab A., Levato R., D’Este M., Piluso S., Eglin D., Malda J. (2020). Printability and Shape Fidelity of Bioinks in 3D Bioprinting. Chem. Rev..

[16] Ribeiro A., Blokzijl M.M., Levato R., Visser C.W., Castilho M., Hennink W.E., Vermonden T., Malda J. (2017). Assessing bioink shape fidelity to aid material development in 3D bioprinting. Biofabrication.

[17] Gonzalez-Fernandez T., Tenorio A.J., Campbell K.T., Silva E.A., Leach J.K. (2021). Alginate-Based Bioinks for 3D Bioprinting and Fabrication of Anatomically Accurate Bone Grafts. Tissue Eng. Part A.

[18] Olate-Moya F., Rubi-Sans G., Engel E., Mateos-Timoneda M.A., Palza H. (2024). 3D Bioprinting of Biomimetic Alginate/Gelatin/Chondroitin Sulfate Hydrogel Nanocomposites for Intrinsically Chondrogenic Differentiation of Human Mesenchymal Stem Cells. Biomacromolecules.

[19] Wang H., Li Q., Jiang Y., Wang X. (2022). Functional Hydrogels with Chondroitin Sulfate Release Properties Regulate the Angiogenesis Behaviors of Endothelial Cells. Gels.

[20] Yang J., Wang S. (2023). Polysaccharide-Based Multifunctional Hydrogel Bio-Adhesives for Wound Healing: A Review. Gels.

[21] Martins E.F., Koba de Moura N., Koba de Moura T., Vasques de Araújo T., Barros Machado J.P., Passador F.R., Esposito E. (2022). Determination and standardization of the kefiran extraction protocol for possible pharmacological applications. Carbohydr. Polym. Technol. Appl..

[22] La Torre C., Fazio A., Caputo P., Tursi A., Formoso P., Cione E. (2022). Influence of Three Extraction Methods on the Physicochemical Properties of Kefirans Isolated from Three Types of Animal Milk. Foods.

[23] Santhamoorthy M., Kim S.-C. (2025). A Review of the Development of Biopolymer Hydrogel-Based Scaffold Materials for Drug Delivery and Tissue Engineering Applications. Gels.

[24] Jalageri M.B., Mohan Kumar G.C. (2022). Hydroxyapatite Reinforced Polyvinyl Alcohol/Polyvinyl Pyrrolidone Based Hydrogel for Cartilage Replacement. Gels.

[25] Uysal B., Madduma-Bandarage U.S.K., Jayasinghe H.G., Madihally S. (2025). 3D-Printed Hydrogels from Natural Polymers for Biomedical Applications: Conventional Fabrication Methods, Current Developments, Advantages, and Challenges. Gels.

[26] Rimada P.S., Abraham A.G. (2003). Comparative study of different methodologies to determine the exopolysaccharide produced by kefir grains in milk and whey. Lait.

[27] Utoiu E., Plangu A., Toma A., Manoiu S.V., Utoiu C.D., Oancea F. (2019). Isolation and characterization of kefiran exopolysaccharides from Romanian kefir grains. Proceedings.

[28] Utoiu E., Oancea A., Stanciuc A.M., Stefan L.M., Toma A., Moraru A., Diguta C.F., Matei F., Cornea C.P., Oancea F. (2018). Prebiotic content and probiotic effect of kombucha fermented pollen. AgroLife Sci. J..

[29] (2009). Biological Evaluation of Medical Devices, Part 5: Tests for In Vitro Cytotoxicity.

[30] Gaspar-Pintiliescu A., Stefan L.M., Mihai E., Sanda C., Manoiu V.S., Berger D., Craciunescu O. (2024). Antioxidant and antiproliferative effect of a glycosaminoglycan extract from Rapana venosa marine snail. PLoS ONE.

[31] Gaspar-Pintiliescu A., Seciu A.M., Miculescu F., Moldovan L., Ganea E., Craciunescu O. (2018). Enhanced extracellular matrix synthesis using collagen dressings loaded with *Artemisia absinthium* plant extract. J. Bioact. Compat. Polym..

[32] Fatma Z.K., Muhammad Y., Ihtesham U.R. (2024). In vitro degradation, swelling, and bioactivity performances of in situ forming injectable chitosan-matrixed hydrogels for bone regeneration and drug delivery. Biotechnol. Bioeng..

